# State- and County-Level Geographic Variation in Opioid Use Disorder, Medication Treatment, and Opioid-Related Overdose Among Medicaid Enrollees

**DOI:** 10.1001/jamahealthforum.2023.1574

**Published:** 2023-06-23

**Authors:** Stephan R. Lindner, Kyle Hart, Brynna Manibusan, Dennis McCarty, K. John McConnell

**Affiliations:** 1 Center for Health Systems Effectiveness, Oregon Health & Science University (OHSU), Portland; 2 OHSU–Portland State University School of Public Health, Portland; 3 Division of General and Internal Medicine, School of Medicine, OHSU, Portland

## Abstract

**Importance:**

The opioid crisis disproportionately affects Medicaid enrollees, yet little systematic evidence exists regarding how prevalence of and health care utilization for opioid use disorder (OUD) vary across geographical areas.

**Objectives:**

To characterize state- and county-level variation in claims-based prevalence of OUD and rates of medication treatment for OUD and OUD-related nonfatal overdose among Medicaid enrollees.

**Design, Setting, and Participants:**

This cross-sectional study used data from the Transformed Medicaid Statistical Information System Analytic Files from January 1, 2016, to December 31, 2018. Participants were Medicaid enrollees with or without OUD in 46 states; Washington, DC; and Puerto Rico who were aged 18 to 64 years and not dually enrolled in Medicare. The analysis was conducted between September 2022 and April 2023.

**Exposure:**

Calendar-year OUD prevalence.

**Main Outcomes and Measures:**

The main outcomes were claims-based measures of OUD prevalence and rates of medication treatment for OUD and opioid-related nonfatal overdose. Individual records were aggregated at the state and county level, and variation was assessed within and across states.

**Results:**

Of the 76 390 817 Medicaid enrollee-year observations included in our study (mean [SD] enrollee age, 36.5 [1.6] years; 59.0% female), 2 280 272 (3.0%) had a claims-based OUD (mean [SD] age, 38.9 [3.6] years; 51.4% female). Of enrollees with OUD, 41.2% were eligible due to Medicaid expansion, 46.4% had other substance use disorders, 55.8% had mental health conditions, 55.2% had claims indicating some form of OUD medication, and 5.8% had claims indicating an overdose during a calendar year. Claims-based outcomes exhibited substantial variation across states: OUD prevalence ranged from 0.6% in Arkansas and Puerto Rico to 9.7% in Maryland, rates of OUD medication treatment ranged from 17.7% in Kansas to 82.8% in Maine, and rates of overdose ranged from 0.3% in Mississippi to 10.5% in Illinois. Pronounced variation was also found within states (eg, OUD prevalence in Maryland ranged from 2.2% in Prince George’s County to 21.6% in Cecil County).

**Conclusions and Relevance:**

In this cross-sectional study of Medicaid enrollees from 2016 to 2018, claims-based prevalence of OUD and rates of OUD medication treatment and opioid-related overdose varied substantially across and within states. Further research appears to be needed to identify important factors influencing this variation.

## Key Points

**Question:**

What is the geographic variation in claims-based prevalence of opioid use disorder (OUD) and rates of OUD medication treatment and OUD-related nonfatal overdose among individuals enrolled in Medicaid from 2016 to 2018?

**Findings:**

In this cross-sectional study of 76 390 817 enrollee-year observations, cross-state OUD prevalence ranged from 0.6% to 9.7%, rates of OUD medication treatment from 17.7% to 82.8%, and rates of OUD-related overdoses from 0.3% to 10.5% across states. Variation was also substantial across counties.

**Meaning:**

The findings suggest that further research is needed to identify factors influencing claims-based OUD prevalence and rates of OUD medication treatment and overdose.

## Teaser

This cross-sectional study analyzes the prevalence of opioid use disorder (OUD) and rates of medication treatment and nonfatal overdose among Medicaid enrollees in the US by state and county from 2016 to 2018.

## Introduction

Opioid use remains an urgent public health problem. In the US in 2019, approximately 10 million people misused opioids, and the number of opioid overdose deaths increased from approximately 51 000 in 2019 to approximately 81 000 in 2021.^1,2,3,4^ The severity of the opioid crisis has varied substantially across the country. For instance, opioid-related overdose deaths per 100 000 population ranged from 10.3 (South Dakota) to 81.4 (Virginia) in 2020.^5^ Opioid prescribing and opioid-related mortality rates have also varied considerably across counties.^6,7,8^

Medicaid is an important payer for opioid use disorder (OUD) treatment, covering close to 40% of people with OUD.^9^ Medicaid enrollees are also disproportionately at risk of an opioid-related overdose.^10^ As a federal-state partnership, Medicaid programs have important differences in terms of coverage for OUD treatment.^9,11,12^ Yet, research on OUD using Medicaid program data has been limited.^13,14^ Our analysis used a newly available national Medicaid claims data source to examine variations in prevalence of OUD and rates of OUD medication treatment and opioid-related overdose at the state and county level.

## Methods

### Data Source

This was a cross-sectional study using Transformed Medicaid Statistical Information System Analytic Files (TAF) data from January 1, 2016, to December 31, 2018.^15^ TAF is a successor to the Medicaid Analytic eXtract file. It includes detailed enrollment and claims information from inpatient, residential, and outpatient settings of both fee-for-service and managed care enrollees. We augmented TAF data with the National Center for Health Statistics (NCHS) 6-level urban-rural classification scheme.^16^ We combined 2016 to 2018 to obtain more precise estimates at the county level and to reduce the likelihood of outliers. The Oregon Health & Science University institutional review board approved the study and granted a waiver of informed consent because data were deidentified. We followed the Strengthening the Reporting of Observational Studies in Epidemiology (STROBE) reporting guideline.

### Study Population

The sample selection approach was guided by our goal to provide a broad overview of the distribution of OUD-related outcomes for the US Medicaid population as measured by TAF data. We included individuals aged 18 to 64 years who were ever enrolled in Medicaid in any of the study years. No enrollment restrictions were applied to the sample. We excluded Medicaid beneficiaries dually enrolled in Medicaid and Medicare and those with missing dual enrollment information because we did not have Medicare claims records. We further excluded individuals with missing county information and individuals residing in counties with fewer than 10 Medicaid enrollees with OUD (identified using *International Statistical Classification of Diseases and Related Health Problems, Tenth Revision [ICD-10]* codes F11.XXX). Finally, we excluded 4 states from the analysis due to data quality concerns. Two of them had fully missing dual eligibility information (Alabama, Utah); the other 2 had fully missing county codes for at least 1 study year (Rhode Island, Wyoming). eAppendixes 1 and 2 in Supplement 1 show details regarding sample selection and data quality considerations.

### Outcomes

We created the following claims-based binary outcome measures at the person-year level: (1) whether a person had an OUD diagnosis during a study year, (2) whether a person with an OUD diagnosis during a study year received some OUD medication during that year, and (3) whether a person with an OUD diagnosis during a study year experienced an opioid-related overdose during that year (eAppendixes 3-5 in Supplement 1). We distinguished between the following types of medication: buprenorphine (including buprenorphine in combination with naloxone), methadone, and naltrexone (oral or extended release). We also calculated a combined measure for whether an enrollee had a claim indicating receipt of any medication for OUD during a calendar year. Procedure codes (all 3 medications) and National Drug Codes from pharmacy claims (buprenorphine and naltrexone) identified medication. We did not use pharmacy claims for methadone because they indicated pain treatment.^13^ We excluded 1 state (Illinois) from analyses involving methadone because the state had no methadone claims despite methadone being a Medicaid benefit and despite opioid treatment programs operating in the state and accepting Medicaid patients during the study period (eAppendix 2 in Supplement 1). *ICD-10* codes (T40.X, X42, X62, and Y12) were used to identify nonfatal opioid-related overdoses; we focused on nonfatal overdoses because fatal overdoses were not well observed in claims records.^17^

### Patient Characteristics

We included the following patient characteristics in our analysis: age, sex, Medicaid eligibility status (adults without disabilities, adults eligible due to Medicaid expansion, adults with disabilities, pregnant women, youths, or unknown), other substance use disorder (*ICD-10* codes F10, F12-F16, F18, F19, F55, 0355, 09931, and 09932), mental health condition (*ICD-10* codes F20-F29, F30-F39, F40-F42, F431, F50, F60, and F9091), and location (large central metropolitan, large fringe metropolitan, medium metropolitan, small metropolitan, micropolitan, noncore, and unknown). We did not include race and ethnicity information due to widespread inaccuracy of these claims as documented by Medicaid’s DQ Atlas.^18^ Specifically, discrepancy in race and ethnicity prevalence exceeded 10% in 22 states in 2018 when comparing TAF with the American Community Survey.

### Data Aggregation

For each outcome, we created a prevalence measure at the county and state level averaging the 3 study years. The numerator was the sum of all enrollee-year observations that had the outcome, and the denominator was the sum of all enrollee-year observations for which the outcome was applicable (all Medicaid enrollees for OUD prevalence; Medicaid enrollees with OUD for all other outcomes). All statistics thus reflected 3-year averages of prevalence for each claims-based outcome. For instance, a person enrolled during the study period with an OUD diagnosis in 2017 and 2018 who started buprenorphine treatment in 2018 would have 3 member-year counts for the OUD prevalence denominator, 2 member-year counts for the denominator of all other outcomes, 2 member-year counts for the OUD prevalence numerator, and 1 member-year count for the numerators of buprenorphine and any OUD medication.

### Statistical Analysis

We first examined the prevalence of outcome values across all enrollee-year observations and for each state separately. We then calculated the following county-level statistics for all outcomes: minimum, first and third quartiles, maximum, IQR, extremal ratio (maximum divided by minimum), and the weighted coefficient of variation (the weighted SD divided by the weighted mean, with weights being the number of enrollees). We also created box and whisker plots for each state’s counties and all counties combined. We examined, for each state, counties with the lowest and highest values.

To assess the amount of heterogeneity at the county and state level, we fitted multilevel mixed-effects logistic regression models for each outcome with random effects for counties nested within random effects for states, weighted by the number of Medicaid enrollees (OUD prevalence) or the number of enrollees with OUD (all other outcomes) residing in each county. We calculated the median odds ratio (MOR), which can be interpreted as the median increase in odds of an outcome if an enrollee moved from a randomly selected area with lower prevalence of the outcome to an area with higher prevalence. Median odds ratio values close to 1.0 indicate low heterogeneity between geographic areas, while higher values indicate more heterogeneity (eAppendix 6 in Supplement 1).

In sensitivity analyses, we (1) calculated rates of treatment for OUD and opioid-related overdose relative to the full Medicaid population and (2) repeated our analysis using a more restrictive set of 30 states based on data quality assessment by DQ Atlas.^18^ All analyses were conducted using R, version 4.1.2 (R Project for Statistical Computing). Data were analyzed between September 2022 and April 2023.

## Results

### Characteristics of the Study Population

The final study population included 76 390 817 Medicaid enrollee-year observations (mean [SD] enrollee age, 36.5 [1.6] years; 59.0% female, 41.0% male) in 2570 counties located in 46 states; Washington, DC; and Puerto Rico (hereafter referred to as states); 2 280 272 (3.0%) had OUD diagnoses (mean [SD] age, 38.9 [3.6] years; 51.4% female, 48.6% male) (Table 1). The largest eligibility groups were adults eligible due to Medicaid expansion (41.2%) and adults without disabilities (33.7%), followed by adults with disabilities (20.5%), pregnant individuals (2.3%), youths (1.2%), and unknown (1.0%). Almost half of the enrollee-year observations (46.4%) had other substance use disorders, and more than half (55.8%) had a mental health condition. Most enrollees lived in metropolitan areas. Compared with the total Medicaid population, those with OUD were younger and more likely to be male, to be part of the expansion group, and to have other substance use disorders or mental health conditions.

**Table 1.** Characteristics of Medicaid Enrollees With OUD

Characteristic	Enrollee-year observations^a^	
	Enrollees with OUD (n = 2 280 272)	All enrollees (N = 76 390 817)
Age, mean (SD), y	38.9 (3.6)	36.5 (1.6)
Sex		
Female	1 172 060 (51.4)	45 070 582 (59.0)
Male	1 108 212 (48.6)	31 320 235 (41.0)
Eligibility group		
Adults eligible due to Medicaid expansion	939 472 (41.2)	28 493 775 (37.3)
Adults with disabilities	467 456 (20.5)	9 625 243 (12.6)
Adults without disabilities	768 452 (33.7)	28 570 166 (37.4)
Pregnant individuals	52 446 (2.3)	2 368 115 (3.1)
Youths	27 363 (1.2)	6 416 829 (8.4)
Unknown	22 803 (1.0)	993 081 (1.3)
Location		
Large central metropolitan	656 718 (28.8)	29 257 683 (38.3)
Large fringe metropolitan	522 182 (22.9)	14 055 910 (18.4)
Medium metropolitan	494 819 (21.7)	15 507 336 (20.3)
Small metropolitan	212 065 (9.3)	6 187 656 (8.1)
Micropolitan	230 307 (10.1)	6 111 265 (8.0)
Noncore	155 058 (6.8)	3 972 322 (5.2)
Unknown	9121 (0.4)	1 298 644 (1.7)
Comorbidities		
Other substance use disorder	1 058 046 (46.4)	4 430 667 (5.8)
Mental health condition	1 272 392 (55.8)	13 903 129 (18.2)

Abbreviation: OUD, opioid use disorder.

^a^ Data are presented as the number (percentage) of enrollee-year observations unless otherwise indicated and are from the Transformed Medicaid Statistical Information System Analytic Files, 2016-2018.^15^

### Variation in OUD Prevalence and Rates of Medication Treatment and Overdose

**Figure 1.**
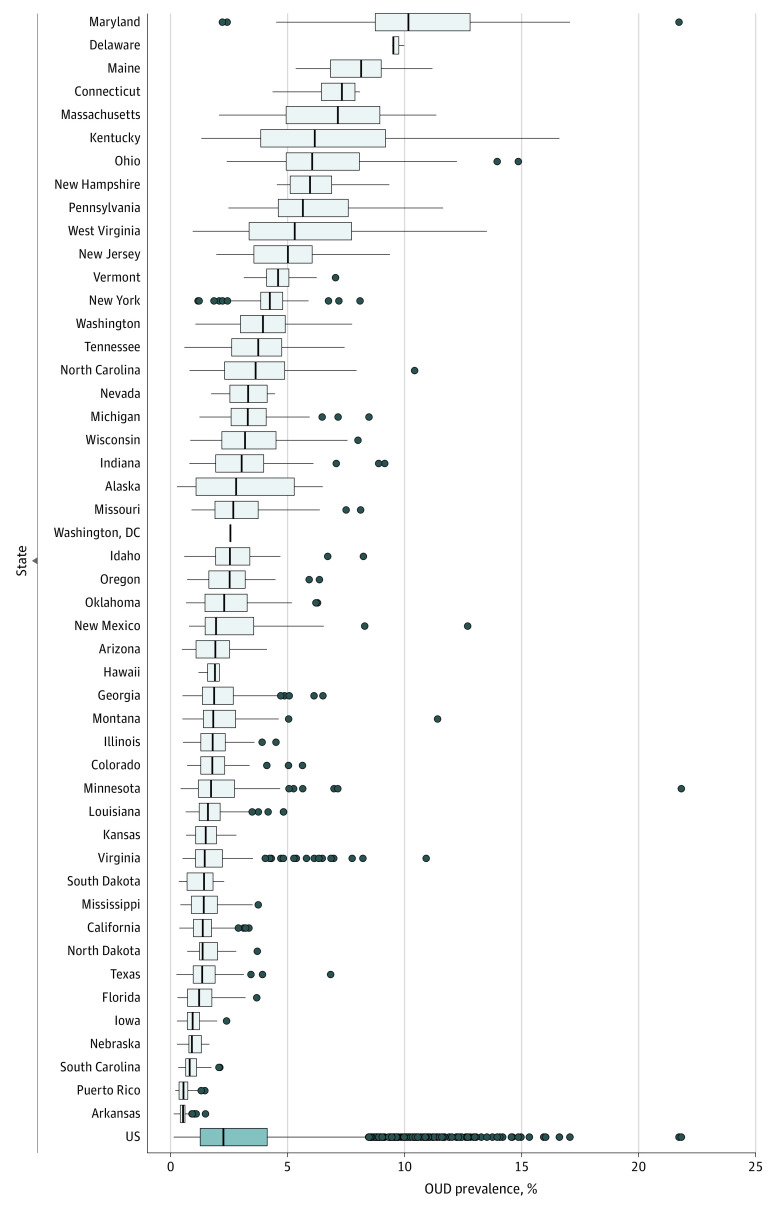
Variation in Opioid Use Disorder (OUD) Prevalence Across and Within States, 2016-2018 Boxes indicate IQRs; vertical lines within boxes, medians; whiskers, 1.5 times the IQR; and dots, outlier counties. No range is displayed for Washington, DC, because it has no counties. Data are from the Transformed Medicaid Statistical Information System Analytic Files, 2016-2018.^15^

The prevalence of claims-based OUD diagnosis varied substantially across and within states (Figure 1, Table 2, and eFigure 1 and eTable 1 in Supplement 1). Maryland (9.7%), Delaware (9.5%), and Maine (8.0%) had more than 2 times the nationwide prevalence of 3.0%. Opioid use disorder diagnostic rates were lowest in Arkansas (0.6%) and Puerto Rico (0.6%).

**Table 2.** Prevalence of Outcomes and Summary Statistics of Variation in Outcomes Across Counties

	OUD	OUD medication					Nonfatal overdose
		Any	Buprenorphine	Methadone	Naltrexone, oral	Naltrexone, extended release	
Prevalence, %							
Overall	2.99	55.24	31.08	22.23	3.05	3.34	5.76
Median (IQR) [range]	2.25 (1.26-4.11) [0.13-21.74]	43.02 (27.63-58.82) [0.00-87.50]	28.94 (17.47-43.99) [0.00-87.50]	2.24 (0.00-14.83) [0.00-82.27]	1.56 (0.00-4.03) [0.00-26.32]	0.31 (0.00-2.96) [0.00-29.42]	3.57 (1.67-5.94) [0.00-25.00]
Extremal ratio^a^	165.42	NA	NA	NA	NA	NA	NA
Coefficient of variation^b^	88.53	32.65	52.82	87.63	82.69	109.89	54.41
Median OR (95% CI)^c^							
County	1.62 (1.60-1.64)	1.68 (1.65-1.71)	1.78 (1.75-1.82)	3.15 (3.00-3.32)	1.61 (1.58-1.65)	1.93 (1.87-2.00)	1.56 (1.53-1.60)
State	1.99 (1.76-2.36)	2.07 (1.82-2.48)	1.82 (1.64-2.11)	36.16 (17.56-107.67)	2.63 (2.20-3.41)	5.66 (4.05-9.14)	1.65 (1.50-1.88)

Abbreviations: NA, not applicable; OR, odds ratio; OUD, opioid use disorder.

^a^ The extremal ratio is the maximum divided by the minimum.

^b^ The weighted coefficient of variation is the weighted SD divided by the weighted mean, with weights being the number of enrollees.

^c^ Median ORs were estimated from multilevel logistic regression models and can be interpreted as the median OR if an enrollee moved from a randomly selected area with lower prevalence to an area with higher prevalence of the outcome.

We also observed substantial variation within states. For instance, claims-based OUD prevalence in Maryland ranged from 2.2% in Prince George’s County to 21.6% in Cecil County (extremal range, 19.4%). States with lower claims-based OUD prevalence tended to have a narrower distribution, with some exceptions (eg, Virginia). Positive outliers beyond 1.5 times the IQR were present in most states and were not an artifact of a small number of Medicaid enrollees. For instance, the minimum number of enrollee-years was 2392 among the 75 counties with an OUD prevalence exceeding 10%.

**Figure 2.**
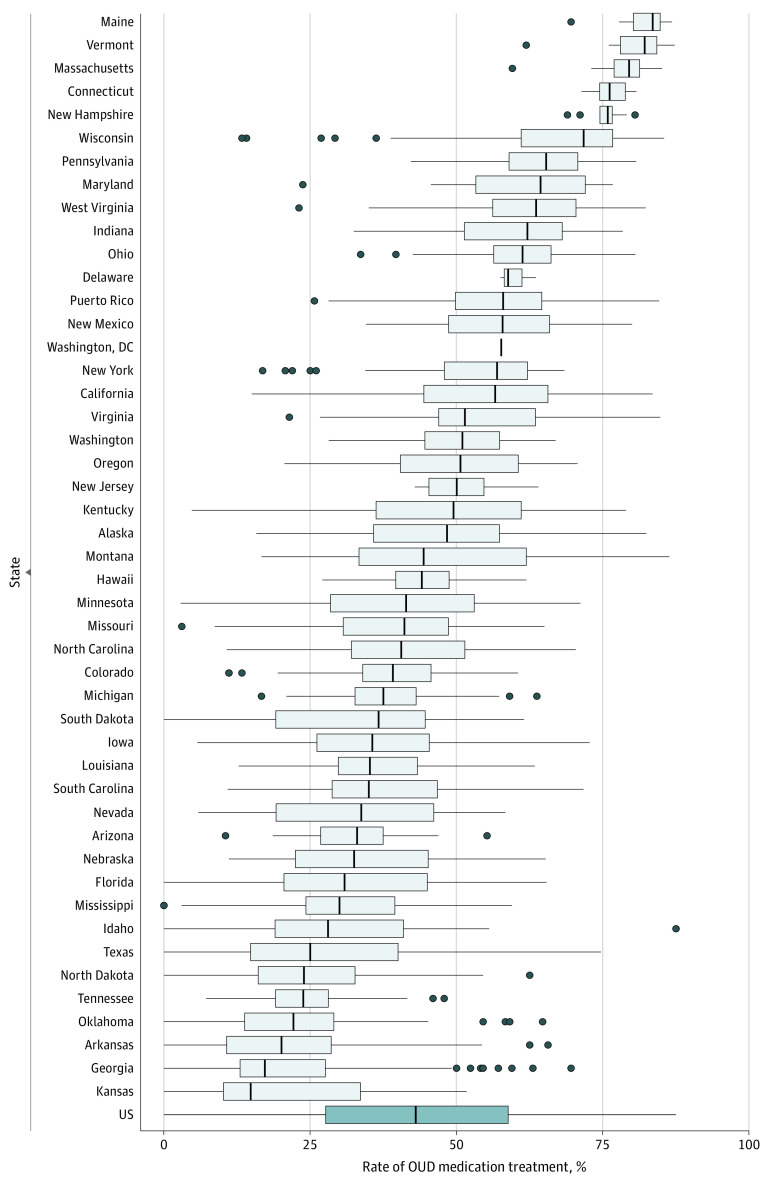
Variation in Rates of Medication Treatment for Opioid Use Disorder (OUD) Across and Within States, 2016-2018 Boxes indicate IQRs; vertical lines within boxes, medians; whiskers, 1.5 times the IQR; and dots, outlier counties. No range is displayed for Washington, DC, because it has no counties. The figure does not include Illinois due to data quality issues. Data are from the Transformed Medicaid Statistical Information System Analytic Files, 2016-2018.^15^

The OUD medication treatment prevalence among Medicaid enrollees with a claims-based OUD diagnosis was 55.2%. Opioid use disorder medication treatment rates ranged from 17.7% in Kansas to 82.8% in Maine (Figure 2 and eFigure 2 and eTable 1 in Supplement 1). There was also substantial variation in claims-based OUD medication treatment rates within states. For instance, we observed 13 states with an IQR of 20% or more, ranging from Oregon (IQR, 40.4%-60.6%) to Montana (IQR, 33.3%-61.9%). Differences in OUD medication treatment rates across states in some cases showed visible discontinuities along state lines (eg, Massachusetts vs New York or North Carolina vs Virginia) (eFigure 2 in Supplement 1).

**Figure 3.**
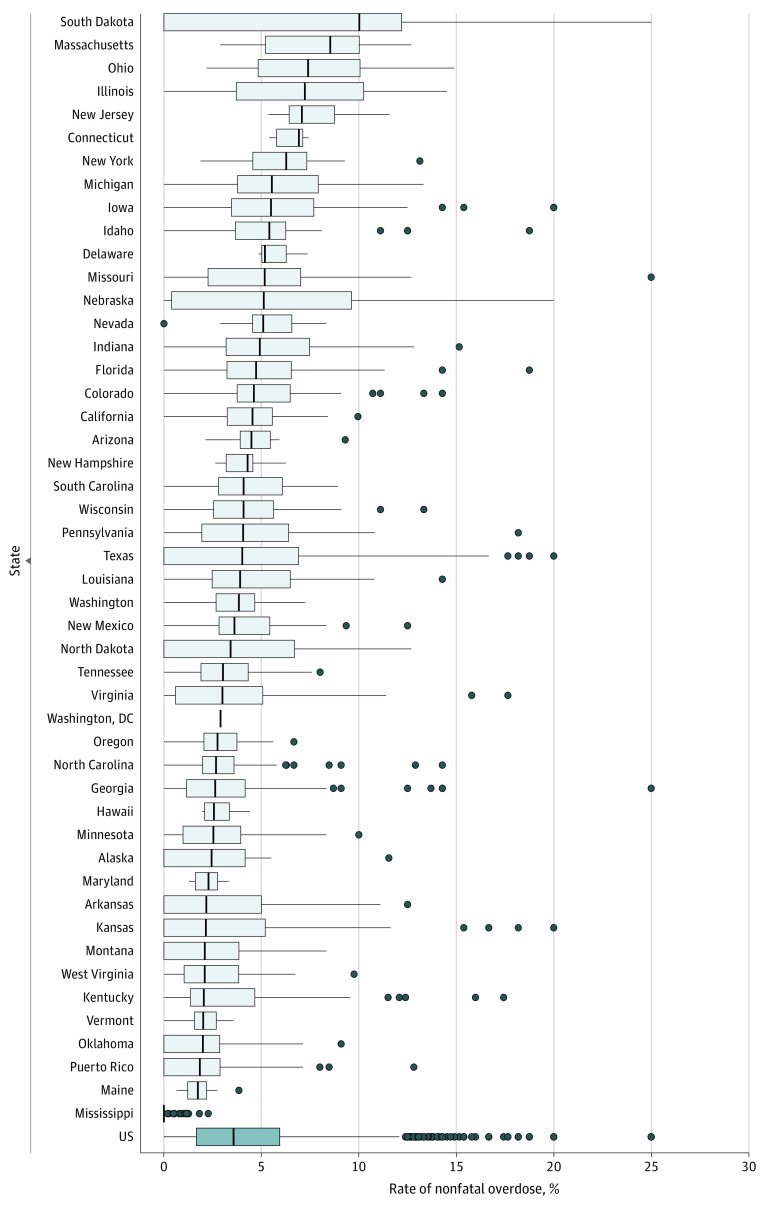
Variation in Rates of Nonfatal Opioid-Related Overdose Across and Within States, 2016-2018 Boxes indicate IQRs; vertical lines within boxes, medians; whiskers, 1.5 times the IQR; and dots, outlier counties. No range is displayed for Washington, DC, because it has no counties. Data are from the Transformed Medicaid Statistical Information System Analytic Files, 2016-2018.^15^

The nationwide prevalence of claims-based overdoses across enrollee-year observations was 5.8%. Claims-based overdose rates ranged from 0.3% in Mississippi to 10.5% in Illinois (Figure 3 and eTable 1 and eFigure 3 in Supplement 1). South Dakota's overdose rate was slightly lower than that in Illinois (10.0%), but its county median overdose rate was the most elevated among all states (10.0%). The distribution of nonfatal overdoses across counties was right-skewed. Substantial overdose levels may have been partially due to small sample sizes: 41 of the 75 counties (54.7%) with an overdose prevalence above 12.0% had fewer than 50 Medicaid enrollee-year observations with OUD. However, 19 of these counties (25.3%) had more than 500 Medicaid enrollee-year observations with OUD, and they were all located in 5 states (Illinois, Kentucky, Massachusetts, Michigan, and Ohio).

### Multilevel Regression Results

County and state MORs were fairly similar for OUD prevalence (county: 1.62 [95% CI, 1.60-1.64]; state: 1.99 [95% CI, 1.76-2.36]) and rates of buprenorphine use (county: 1.78 [95% CI, 1.75-1.82]; state: 1.82 [95% CI, 1.64-2.11]) and nonfatal overdose (county: 1.56 [95% CI, 1.53-1.60]; state: 1.65 [95% CI, 1.50-1.88]) (Table 2), with the state MORs being slightly larger than the county MORs. The state MOR was larger for naltrexone (oral: 2.63 [95% CI, 2.20-3.41]; extended-release: 5.66 [95% CI, 4.05-9.14]) and methadone (36.16 [95% CI, 17.56-107.67]) than the county MOR possibly due to state-level variation in medication coverage.

### Sensitivity Analysis

Opioid use disorder medication rates relative to the total Medicaid population ranged from 0.1% in Arkansas to 6.6% in Maine (eFigure 4 in Supplement 1). Ranking of states by OUD medication was similar to that in the main analysis, with Puerto Rico being the exception due to its low OUD prevalence rate.

Nonfatal overdose rates in the total Medicaid population ranged from 4.9 per 100 000 population in Mississippi to 607.0 per 100 000 population in Delaware (eFigure 5 in Supplement 1). South Dakota was among the states with a low rate of nonfatal overdoses relative to the Medicaid population in the state (80.3 per 100 000 population) despite a substantial overdose rate compared with individuals with OUD, which was due to its low OUD prevalence rate.

Statistics for the distribution of outcomes were similar for the restricted sample of 30 states (eTable 2 in Supplement 1). The only notable difference was for methadone treatment, which was more prevalent and had a lower MOR in the restricted sample. The restricted sample excluded some states with low OUD prevalence and data quality concerns related to *ICD-10* codes (eg, Puerto Rico, South Carolina).

## Discussion

This cross-sectional study of Medicaid claims data from 2016 to 2018 found substantial variation in claims-based prevalence of OUD and rates of medication treatment for OUD and opioid-related overdose across and within states. There are a number of factors that may explain the variation, which may inform future research. First, claims-based measures of OUD, receipt of OUD medication treatment, and OUD-related overdoses depend on whether patients with OUD seek and are able to access health care and how health care professionals interact with these patients. Personal barriers (eg, stigma, shame, and lack of trust in the health care system) and structural barriers (eg, lack of available health care professionals, lack of OUD-related training by health care professionals) may affect whether a patient with OUD has an OUD diagnosis in Medicaid claims, engages in medication treatment, and is admitted to an emergency department in case of an overdose.^19^ Future research could investigate the role of such barriers for claims-based prevalence of OUD, OUD medication treatment, and overdoses. Based on our analysis, 1 option for such research might be to compare adjacent counties from different states for which there are discontinuities at state lines. Second, rates of OUD, OUD medication treatment, and overdose across and within states may be affected by state policies. Examples of such policies include Medicaid reimbursement rates that affect health care professionals’ willingness to see Medicaid patients; educational outreach to primary care physicians, psychologists, nurses, and other health care professionals about the importance of diagnosing OUD and the effectiveness of OUD medication; and care coordination efforts to sustain OUD medication treatment when patients move from 1 treatment setting to another (eg, from intensive residential treatment settings to outpatient settings). Integrating such policies into TAF claims records and identifying policy changes or other natural experiments to identify the effects of such policies may be focuses for future research. Third, some variation may be related to patient characteristics. Assessing the role of demographic and other characteristics may be an important future research topic. Fourth, variation in outcomes might in some instances reflect missing TAF records due to data quality issues. Data missing completely at random would not bias prevalence levels, but nonrandom missing records could lead to overestimation or underestimation of outcomes. Research benchmarking TAF records against more complete state Medicaid claims records may provide insight about the implications of incomplete TAF records.

The overall rate of OUD medication use was 55.2%, consistent with Medicaid claims-based evidence from 11 northeastern states, which reported an increase in medication treatment among Medicaid enrollees with OUD from 47.8% in 2014 to 57.1% in 2018.^13^ However, both that study and ours used Medicaid claims records and thus only measured medication treatment rates among Medicaid enrollees who interacted with the health care system. Studies using the National Survey on Drug Use and Health reported OUD medication treatment rates in the range of 11% to 15% for the same period.^20,21^ These results suggest underreporting of OUD diagnoses in Medicaid claims. Understanding characteristics of Medicaid enrollees with undiagnosed OUD in Medicaid claims and factors contributing to such underreporting may be important topics for future research.

In this study, OUD medication treatment rates were below 50% in some states. Methadone dispensing, in particular, varied substantially across states, partially because some states did not cover methadone during our study period. However, methadone dispensing was low even among states that covered it. Further improving access to all types of medication for OUD should remain an important goal for state Medicaid programs.

### Limitations

This study has several limitations. Fatal overdoses were not well observed in our data, likely because many of these occurred in nonmedical settings or were not associated with a medical intervention that resulted in a claim.^17^ Connecting TAF records to National Death Index records from the NCHS would significantly improve mortality information in Medicaid data. However, the Centers for Medicare & Medicaid Services currently does not provide personal identifiers to the NCHS on behalf of researchers to enable this linkage. Changes in this policy could open avenues to research that provides direct public health benefits. We also observed no methadone dispensing in 1 state that covered this treatment. This finding might reflect data quality issues or may be related to specific payment procedures that we could not capture. Using claims records to identify people with OUD may result in measurement errors that could have led to an underestimation of OUD prevalence.^13,22,23^ Finally, our study was limited by small sample sizes for some counties. We sought to address this concern by excluding counties with low sample sizes, combining 3 years of data, and adjusting outcome values.

## Conclusions

In this cross-sectional study of 76 390 817 Medicaid enrollee-years, 2 280 272 of which had an OUD diagnosis, claims-based prevalence of OUD and rates of OUD medication treatment and opioid-related overdose varied substantially across and within states. Future research may leverage this information to identify important factors influencing these outcomes.

## Notes

Supplement 1. **eAppendix 1.** Consort Diagram **eAppendix 2.** Data Quality Assessment and Exclusion of States **eAppendix 3.** Diagnosis for Opioid Use Disorder Measure **eAppendix 4.** Medication for Opioid Use Disorder Measure **eAppendix 5.** Nonfatal Opioid-Related Overdoses in Clinical Settings Measure **eAppendix 6.** Description of Mixed-Effects Multilevel Logistic Regression Model **eFigure 1.** Geographical Distribution of Opioid Use Disorder Prevalence **eFigure 2.** Geographical Distribution of Opioid Use Disorder Medication Treatment **eFigure 3.** Geographical Distribution of Nonfatal Overdoses **eFigure 4.** Geographical Distribution of Opioid Use Disorder Medication Treatment Using All Medicaid Enrollees as Denominator **eFigure 5.** Geographical Distribution of Nonfatal Overdoses Using All Medicaid Enrollees as Denominator **eTable 1.** Overall and State-level Prevelance Levels of Opioid Use Disorder Diagnosis, Medication, and Nonfatal Overdoses **eTable 2.** Distribution of Outcomes, Restricted Sample
eReferences

Supplement 2.
Data Sharing Statement

## References

[1] Substance Abuse and Mental Health Services Administration. Key Substance Use and Mental Health Indicators in the United States: Results From the 2019 National Survey on Drug Use and Health. Substance Abuse and Mental Health Services Administration; 2020. 39437027

[2] Hedegaard H, Miniño AM, Warner M. Drug Overdose Deaths in the United States, 1999-2019. National Center for Health Statistics; 2020.

[3] Centers for Disease Control and Prevention. Drug overdose deaths in the US up 30% in 2020. July 14, 2021. Accessed April 5, 2023. https://www.cdc.gov/nchs/pressroom/nchs_press_releases/2021/20210714.htm

[4] Centers for Disease Control and Prevention. US overdose deaths in 2021 increased half as much as in 2020—but are still up 15%. May 11, 2022. Accessed April 5, 2023. https://www.cdc.gov/nchs/pressroom/nchs_press_releases/2022/202205.htm

[5] Centers for Disease Control and Prevention. Drug overdose mortality by state. 2022. Accessed April 5, 2023. https://www.cdc.gov/nchs/pressroom/sosmap/drug_poisoning_mortality/drug_poisoning.htm

[6] Guy GP Jr, Zhang K, Schieber LZ, Young R, Dowell D. County-level opioid prescribing in the United States, 2015 and 2017. JAMA Intern Med. 2019;179(4):574-576. doi:10.1001/jamainternmed.2018.6989 30742206 PMC6450301

[7] Langabeer JR, Chambers KA, Cardenas-Turanzas M, Champagne-Langabeer T. County-level factors underlying opioid mortality in the United States. Subst Abus. 2022;43(1):76-82. doi:10.1080/08897077.2020.1740379 32186475

[8] Monnat SM, Peters DJ, Berg MT, Hochstetler A. Using Census data to understand county-level differences in overall drug mortality and opioid-related mortality by opioid type. Am J Public Health. 2019;109(8):1084-1091. doi:10.2105/AJPH.2019.305136 31219718 PMC6611117

[9] Oregera K, Tolbert J. The opioid epidemic and Medicaid’s role in facilitating access to treatment. Kaiser Family Foundation. May 24, 2019. Accessed May 10, 2023. https://www.kff.org/medicaid/issue-brief/the-opioid-epidemic-and-medicaids-role-in-facilitating-access-to-treatment/

[10] Weiner SG, El Ibrahimi S, Hendricks MA, . Factors associated with opioid overdose after an initial opioid prescription. JAMA Netw Open. 2022;5(1):e2145691. doi:10.1001/jamanetworkopen.2021.45691 35089351 PMC8800077

[11] Grogan CM, Andrews C, Abraham A, . Survey highlights differences in Medicaid coverage for substance use treatment and opioid use disorder medications. Health Aff (Millwood). 2016;35(12):2289-2296. doi:10.1377/hlthaff.2016.0623 27920318 PMC5304419

[12] Andrews CM, Grogan CM, Westlake MA, . Do benefits restrictions limit Medicaid acceptance in addiction treatment? results from a national study. J Subst Abuse Treat. 2018;87:50-55. doi:10.1016/j.jsat.2018.01.010 29471926 PMC5826552

[13] Donohue JM, Jarlenski MP, Kim JY, ; Medicaid Outcomes Distributed Research Network (MODRN). Use of medications for treatment of opioid use disorder among US Medicaid enrollees in 11 states, 2014-2018. JAMA. 2021;326(2):154-164. doi:10.1001/jama.2021.7374 34255008 PMC8278273

[14] McCarty D, Gu Y, McIlveen JW, Lind BK. Medicaid expansion and treatment for opioid use disorders in Oregon: an interrupted time-series analysis. Addict Sci Clin Pract. 2019;14(1):31. doi:10.1186/s13722-019-0160-6 31416475 PMC6694675

[15] Chronic Conditions Warehouse. Chronic Conditions Warehouse User Guide: T-MSIS Analytic Files (TAF) Research Identifiable Files (RIFS). Chronic Conditions Warehouse; 2019.

[16] Ingram DD, Franco SJ. NCHS urban-rural classification scheme for counties. Vital Health Stat 2. 2012;(154):1-65. 22783637

[17] Nguyen JK, Sanghavi P. A national assessment of legacy versus new generation Medicaid data. Health Serv Res. 2022;57(4):944-956. doi:10.1111/1475-6773.13937 35043402 PMC9264472

[18] Medicaid.gov. DQ Atlas. Accessed April 5, 2023. https://www.medicaid.gov/dq-atlas/

[19] Priester MA, Browne T, Iachini A, Clone S, DeHart D, Seay KD. Treatment access barriers and disparities among individuals with co-occurring mental health and substance use disorders: an integrative literature review. J Subst Abuse Treat. 2016;61:47-59. doi:10.1016/j.jsat.2015.09.006 26531892 PMC4695242

[20] Krawczyk N, Rivera BD, Jent V, Keyes KM, Jones CM, Cerdá M. Has the treatment gap for opioid use disorder narrowed in the US? a yearly assessment from 2010 to 2019. Int J Drug Policy. 2022;110:103786. doi:10.1016/j.drugpo.2022.103786 35934583 PMC10976290

[21] Substance Abuse and Mental Health Services Administration. *Key Substance Use and Mental Health Indicators in the United States: Results From the 2018 National Survey on Drug Use and Health*. HHS Publication PEP19-5068, NSDUH Series H-54. Substance Abuse and Mental Health Services Administration; 2019.

[22] Carrell DS, Albertson-Junkans L, Ramaprasan A, . Measuring problem prescription opioid use among patients receiving long-term opioid analgesic treatment: development and evaluation of an algorithm for use in EHR and claims data. J Drug Assess. 2020;9(1):97-105. doi:10.1080/21556660.2020.1750419 32489718 PMC7241518

[23] Howell BA, Abel EA, Park D, Edmond SN, Leisch LJ, Becker WC. Validity of incident opioid use disorder (OUD) diagnoses in administrative data: a chart verification study. J Gen Intern Med. 2021;36(5):1264-1270. doi:10.1007/s11606-020-06339-3 33179145 PMC8131432

