# Supplementary material for: State- and County-Level Geographic Variation in Opioid Use Disorder, Medication Treatment, and Opioid-Related Overdose Among Medicaid Enrollees
Source: JAMA Health Forum. 2023 Jun 23;4(6):e231574. doi: 10.1001/jamahealthforum.2023.1574 (PMC10290243; doi:10.1001/jamahealthforum.2023.1574)
Supplement: Supplement 1. — eAppendix 1. Consort Diagram eAppendix 2. Data Quality Assessment and Exclusion of States eAppendix 3. Diagnosis for Opioid Use Disorder Measure eAppendix 4. Medication for Opioid Use Disorder Measure eAppendix 5. Nonfatal Opioid-Related Overdoses in Clinical Settings Measure eAppendix 6. Description of Mixed-Effects Multilevel Logistic Regression Model eFigure 1. Geographical Distribution of Opioid Use Disorder Prevalence eFigure 2. Geographical Distribution of Opioid Use Disorder Medication Treatment eFigure 3. Geographical Distribution of Nonfatal Overdoses eFigure 4. Geographical Distribution of Opioid Use Disorder Medication Treatment Using All Medicaid Enrollees as Denominator eFigure 5. Geographical Distribution of Nonfatal Overdoses Using All Medicaid Enrollees as Denominator eTable 1. Overall and State-level Prevelance Levels of Opioid Use Disorder Diagnosis, Medication, and Nonfatal Overdoses eTable 2. Distribution of Outcomes, Restricted Sample eReferences [file jamahealthforum-e231574-s001.pdf]

Supplementary Online Content

Lindner SR, Hart K, Manibusan B, McCarty D, McConnell KJ. State- and county-level geographic variation in opioid use disorder, medication treatment, and opioid-related overdose among Medicaid enrollees. *JAMA Health Forum*. 2023;4(6):e231574. doi:10.1001/jamahealthforum.2023.1574

eAppendix 1. Consort Diagram

eAppendix 2. Data Quality Assessment and Exclusion of States

eAppendix 3. Diagnosis for Opioid Use Disorder Measure

eAppendix 4. Medication for Opioid Use Disorder Measure

eAppendix 5. Nonfatal Opioid-Related Overdoses in Clinical Settings Measure

eAppendix 6. Description of Mixed-Effects Multilevel Logistic Regression Model

eFigure 1. Geographical Distribution of Opioid Use Disorder Prevalence

eFigure 2. Geographical Distribution of Opioid Use Disorder Medication Treatment

eFigure 3. Geographical Distribution of Nonfatal Overdoses

eFigure 4. Geographical Distribution of Opioid Use Disorder Medication Treatment Using All Medicaid Enrollees as Denominator

eFigure 5. Geographical Distribution of Nonfatal Overdoses Using All Medicaid Enrollees as Denominator

eTable 1. Overall and State-level Prevelance Levels of Opioid Use Disorder Diagnosis, Medication, and Nonfatal Overdoses

eTable 2. Distribution of Outcomes, Restricted Sample

eReferences

This supplementary material has been provided by the authors to give readers additional information about their work.

© 2023 Lindner SR et al. *JAMA Health Forum.*

eAppendix 1. Consort diagram

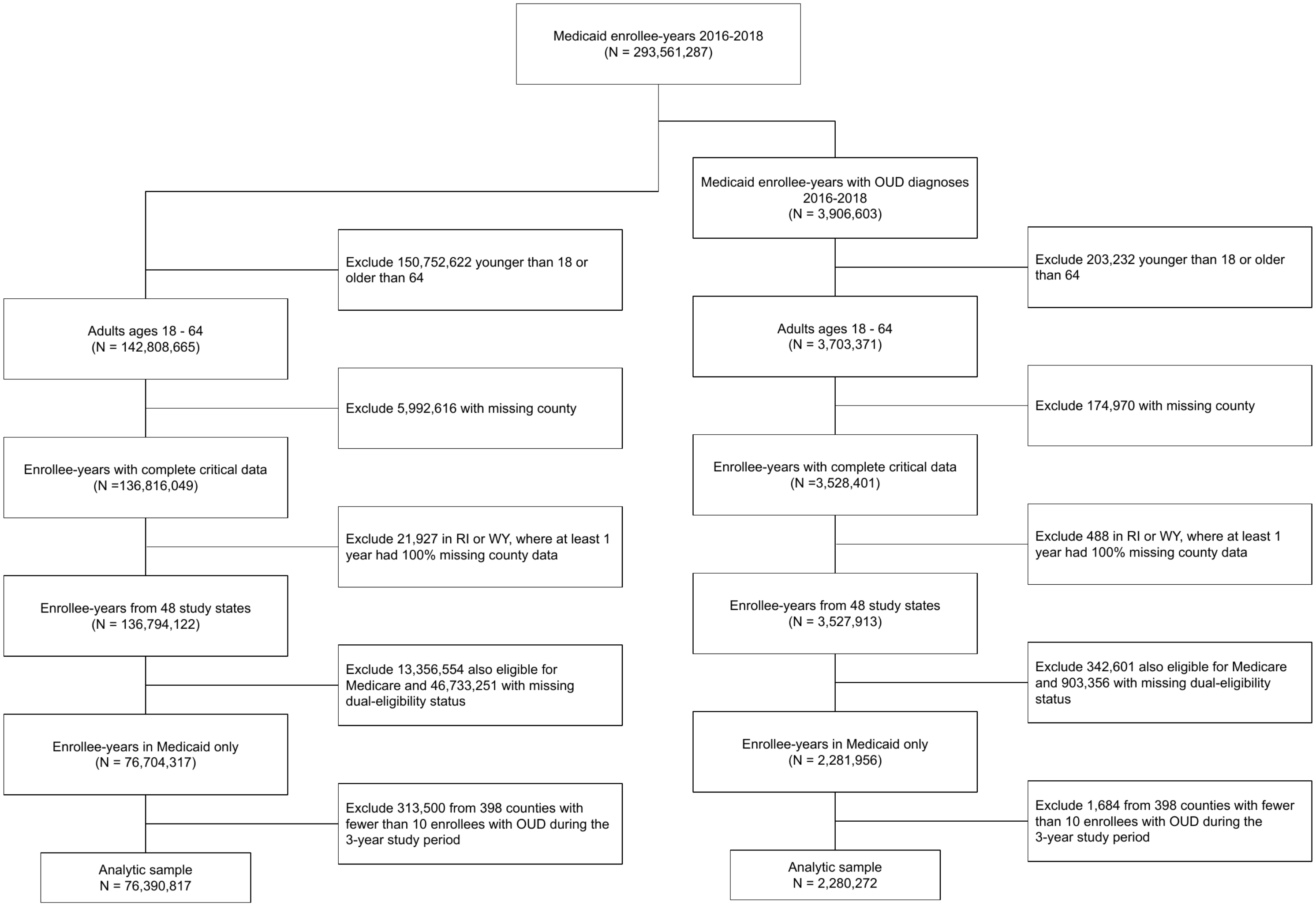

© 2023 Lindner SR et al. *JAMA Health Forum.*

eAppendix 2. Data quality assessment and exclusion of states

We selected two sets of states based on data quality assessment for our study.

Exclusion of states for the main analysis. Our intention for the main analysis was to provide a full overview of the distribution of TAF data. To this end, we only applied minimal exclusion criteria based on data quality assessment. Specifically, we excluded the following states from our analysis that had completely missing dual eligibility or county information for at least one study year:

- Rhode Island (missing county information in 2017, 2018);
- Wyoming (missing county information in 2016);
- Alabama (missing dual eligibility information, 2016-2018);
- Utah (missing dual eligibility information, 2016-2018).

We also excluded one state (Illinois) from analyses related to methadone treatment or any medication for OUD treatment because the state had zero methadone claims, at least one Opioid Treatment Program (OTP) accepting Medicaid patients operating in that year (based on publicly available information about OTPs from the National Survey of Substance Abuse Treatment Services, N-SSATS), and covered methadone 2016-2018 based on our review of state policies.[1-4] Thus, we would expect to observe methadone claims in Illinois, but did not.

Exclusion of states for sensitivity analysis. We created a more restrictive sample of states based on data quality assessment by the DQ Atlas.[5] DQ Atlas classified TAF data elements from "low concern" to "unusable" based, for data elements relevant to our analysis, on expected completeness of data elements. The exact data quality assessment depended on the type of claims. For instance, professional claims in TAF other service records should always have a procedure code, and DQ Atlas thus examined the fraction of claims with missing procedure codes to classify states' data quality for these claims. The DQ Atlas did not assess completeness of county codes, but it assessed completeness of zip codes. We categorized states' data quality with respect to completeness of county codes based on the same criteria that DQ Atlas uses for zip codes. There is currently no data quality assessment for NDC codes, so OUD medication data quality assessment was only based on HCPCS codes.

© 2023 Lindner SR et al. *JAMA Health Forum.*

The following table shows states we excluded from the sensitivity analysis as well as data elements and years categorized as “high concern” or unusable.” For example, Colorado was excluded from the sensitivity analysis because it had HCPCS codes with such data quality assessment for the year 2016. In total, we included 30 states and excluded 22 states from the sensitivity analysis.

© 2023 Lindner SR et al. *JAMA Health Forum.*

State	ICD codes: OUD diagnosis / overdose)	HCPCS codes: Medication for OUD	Enrollment records: Dual eligibility	Enrollment records: county codes
Alabama			2016-2018	
Arkansas			2016	
Colorado		2016		
Georgia		2016-2018		
Illinois	2016	2016-2018		
Kentucky		2016		
Maryland	2016-2017	2016-2018		
Michigan			2016	
Nebraska				2016
Nevada		2016		2016
New Hampshire	2016-2018			
New York		2016-2018		
North Dakota				2017-2018
Pennsylvania		2016-2018		2016-2017
Puerto Rico	2016-2018	2016-2018		
Rhode Island				2016-2018
South Carolina	2016-2018	2016-2018		
Tennessee	2017-2018			
Texas	2018	2016-2018		2016
Utah		2016-2018	2016-2018	

© 2023 Lindner SR et al. *JAMA Health Forum.*

Vermont	2016-2018
Wyoming	2016

© 2023 Lindner SR et al. *JAMA Health Forum.*

eAppendix 3. Diagnosis for opioid use disorder (OUD) measure

Denominator: Medicaid-enrolled individuals ages 18 to 64 not dually enrolled in Medicare for at least one month during a calendar year.

Numerator: Whether an individual included in the denominator had at least one OUD diagnosis during the calendar year. Diagnosis codes used to identify individuals with OUD included:

1. ICD-9: 30400, 30401, 30402, 30470, 30471, 30472, 30550, 30551, 30552

2. ICD-10: F1110, F11120, F11121, F11122, F11129, F1114, F11150, F11151, F11159, F11181, F11182, F11188, F1119, F1120, F11220, F11221, F11222, F11229, F1123, F1124, F11250, F11251, F11259, F11281, F11282, F11288, F1129

© 2023 Lindner SR et al. *JAMA Health Forum.*

eAppendix 4. Medication for OUD measure

Denominator: Medicaid-enrolled individuals ages 18 to 64 not dually enrolled in Medicare.

Numerator: Whether an individual included in the denominator had at least one claim matching one of the codes for medications for OUD.

Medication	Code type	Codes
Buprenorphine	NDC	63481016101, 63481068501, 50383092493, 49999063830, 63481016160, 63481068560, 00093537956, 50383093093, 49999063930, 63481020701, 63481082001, 00228315303, 55700030230, 63874117303, 63481020760, 63481082060, 00228315603, 55700030330, 63481034801, 63481095201, 68308020230, 63481034860, 63481095260, 68308020830, 63481051901, 00054017613, 35356055530, 12496127802, 63481051960, 00054017713, 35356055630, 12496131002
Buprenorphine / Naloxone	NDC	59385001201, 00093572056, 42291017430, 12496120401, 63629403401, 63629403402, 54123011430, 59385001601, 00228315503, 54569640800, 12496120803, 16590066630, 52959030430, 54868570702, 63629403403, 54123091430, 59385001630, 00228315573, 55700018430, 35356000407, 52959074930, 54868570703, 63874108403, 54123092930, 00054018813, 00406192303, 65162041503, 12496121203, 35356000430, 54569549600, 54868570704, 63874108503, 54123095730, 00054018913, 00406192403, 65162041603,

© 2023 Lindner SR et al. *JAMA Health Forum.*

		43063018407, 54569573900, 54868575000, 66336001630, 54123098630, 54569639900, 43063018430, 54569573901, 55045378403, 68071138003, 59385001230, 00093572156, 42291017530, 12496120203, 55700014730, 49999039507, 54569573902, 63629402801, 68071151003, 59385001401, 00228315403, 50383028793, 12496128302, 49999039515, 54868570700, 68258299903, 12496120403, 59385001430, 00228315473, 50383029493, 12496120801, 12496130602, 49999039530, 54868570701
Buprenorphine	HCPCS	J0571, J0572, J0573, J0574, J0575
Naltrexone	NDC	00406009201, 16729008101, 68084029121, 51224020650, 00406117003, 00555090201, 51285027502, 00406009203, 16729008110, 52152010502, 68094085362, 00185003901, 42291063230, 52152010504, 68115068030, 00185003930, 43063059115, 52152010530, 00056001122, 00406117001, 47335032683, 54868557400, 00056001130, 47335032688, 65694010003, 00056001170, 50436010501, 65694010010, 00056007950, 00555090202, 51224020630, 68084029111, 51285027501
Naltrexone, extended-release injectable	NDC	63459030042, 65757030001, 65757030202

© 2023 Lindner SR et al. *JAMA Health Forum.*

Naltrexone, extended-release injectable	HCPCS	J2315, T1502
Methadone administration	HCPCS	H0020, S0109

© 2023 Lindner SR et al. *JAMA Health Forum.*

eAppendix 5. Non-fatal opioid-related overdoses in clinical settings measure

Denominator: Medicaid-enrolled individuals ages 18 to 64 not dually enrolled in Medicare.

Numerator: Whether an individual included in the denominator had at least one claim matching one of the codes for opioid-related overdoses:

1. ICD-9: 965.00-965.02, 965.09, E850.0-E850.2

2. ICD-10: T40.0-T40.4, X42, X62, Y12

© 2023 Lindner SR et al. *JAMA Health Forum.*

eAppendix 6. Description of mixed-effects multi-level logistic regression model

To characterize heterogeneity at county and state levels, we fitted mixed-effects multi-level logistic regression models for each outcome with random effects for counties nested within states. We used logistic regression models because all outcomes were binary. The model specification was as follows:

$$logit\left(p_{ics} = P(y_{ics} = 1)\right)$$
$$log\left(\frac{p_{ics}}{1 - p_{ics}}\right) = \beta_0 + \alpha_s + b_c$$
$$\alpha_s \sim N(0, \sigma_s^2), b_c \sim N(0, \sigma_c^2)$$
$$i = 1,2, \dots, n_c c = 1,2, \dots, n_s s = 1,2, \dots, S$$

where $p_{ics} = P(y_{ics} = 1)$ is the probability that individual i experiences the outcome y, and β_0 is the overall mean prevalence of the outcome expressed on the logistic scale. The random effect α_s is the state-level residual error, which represents the smoothed difference between the population-level prevalence and the prevalence in state s. The random effect b_c is nested within the state-level random effect and represents the smoothed difference between the state-level prevalence and county-level prevalence.

In the case of a continuous outcome fitted to a linear mixed-effects multi-level model, the intraclass correlation coefficient (ICC) is commonly used to characterize heterogeneity at different levels. However, this statistic is difficult to interpret in the case of logistic regression models applied to binary outcomes, because there is no estimate of individual-level residual error in these models (the variance of a binomial distribution is a function of the mean p only). Alternative approaches to calculating ICC in the case of logistic regression models often depend on the overall prevalence of the outcome, so ICCs for models of outcomes with differing baseline prevalences cannot be compared. Also, the higher-level variance derived from the random effects is on the logit scale, whereas the individual-level variance is on the probability scale, so the two measures are not directly comparable.

For these reasons, we calculated the Median Odds Ratio (MOR), a commonly used approach for measuring the effect of clustering in multi-level logistic regression models. Conceptually, the MOR is derived by calculating the odds ratio for every possible combination of two of the regions contained in a random effect, where the region with the higher prevalence of the outcome is always placed in the numerator. The median of these odds ratios at that level of regional area is then defined as the MOR. The MOR can be interpreted as

© 2023 Lindner SR et al. *JAMA Health Forum.*

the median increase in odds of an outcome if an enrollee moved from a randomly selected area with lower prevalence of the outcome to an area with higher prevalence. By construction, the MOR is always 1.0 or higher, with values closer to 1.0 implying less variance at the regional level, and larger values indicating more heterogeneity. The MOR has the advantage of being independent of baseline prevalences and can be interpreted on exactly the same scale as any other odds ratio.

© 2023 Lindner SR et al. *JAMA Health Forum.*

eFigure 1. Geographical distribution of OUD prevalence

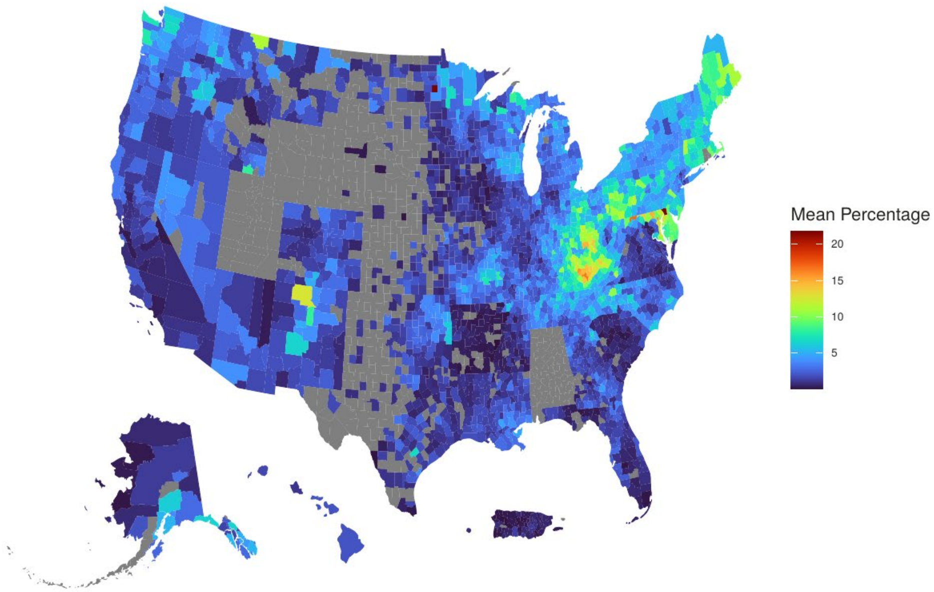

Source: Transformed Medicaid Statistical Information System (T-MSIS) Analytic Files (TAF), 2016-2018

© 2023 Lindner SR et al. *JAMA Health Forum.*

eFigure 2. Geographical distribution of OUD medication treatment

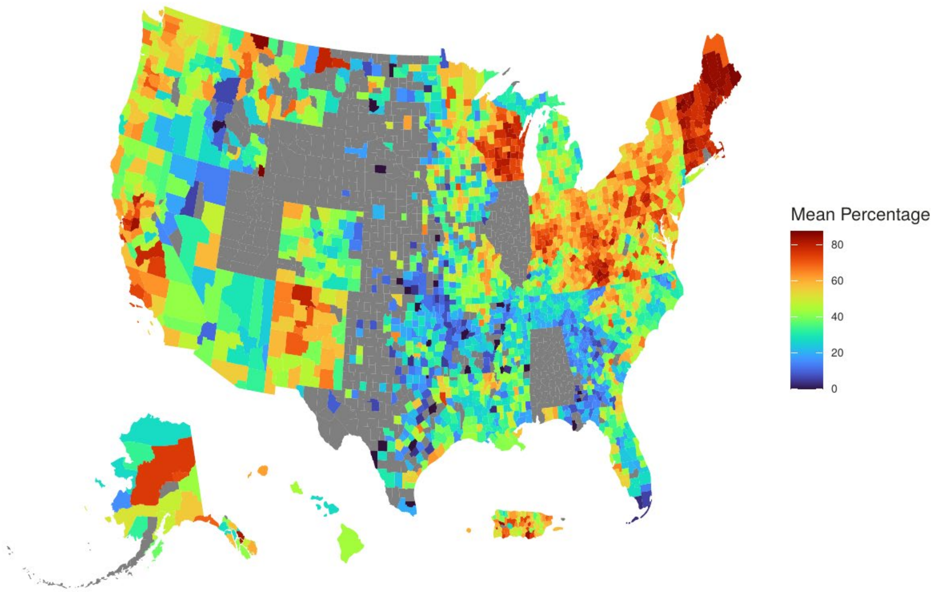

Source: Transformed Medicaid Statistical Information System (T-MSIS) Analytic Files (TAF), 2016-2018

© 2023 Lindner SR et al. *JAMA Health Forum.*

eFigure 3. Geographical distribution of non-fatal overdoses

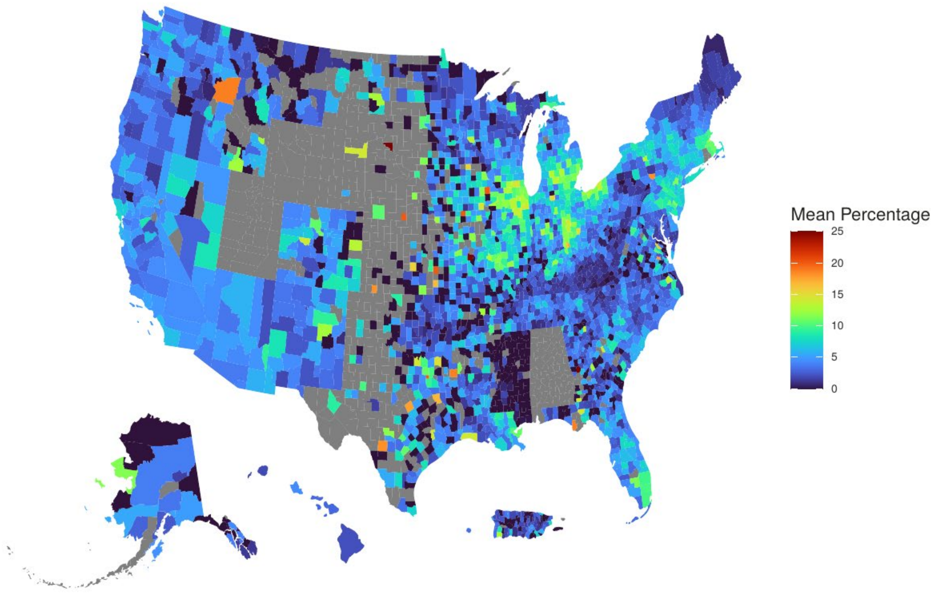

Source: Transformed Medicaid Statistical Information System (T-MSIS) Analytic Files (TAF), 2016-2018

© 2023 Lindner SR et al. *JAMA Health Forum.*

eFigure 4. Geographical distribution of OUD medication treatment (using all Medicaid enrollees as denominator)

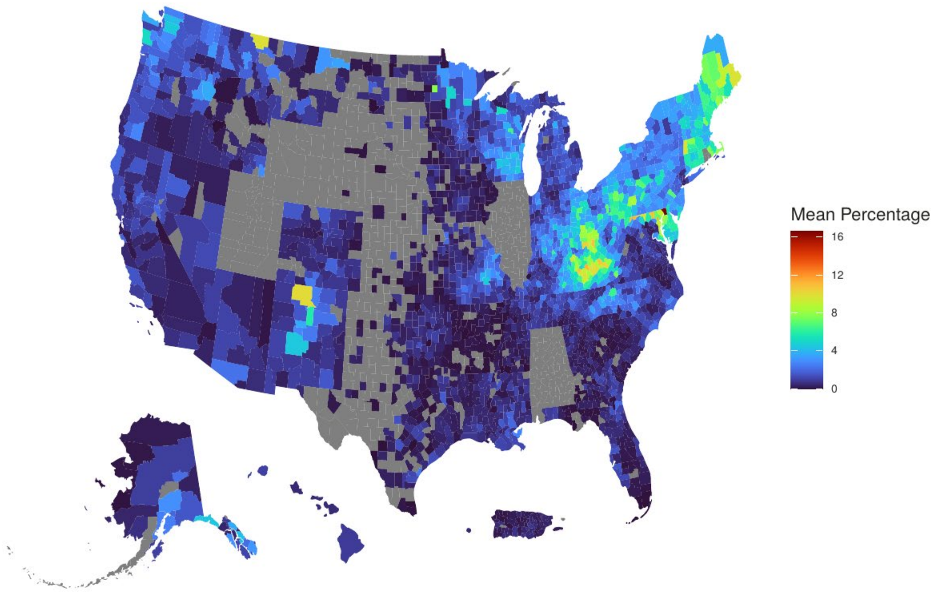

Source: Transformed Medicaid Statistical Information System (T-MSIS) Analytic Files (TAF), 2016-2018

© 2023 Lindner SR et al. *JAMA Health Forum.*

eFigure 5. Geographical distribution of non-fatal overdoses (using all Medicaid enrolleees as denominator)

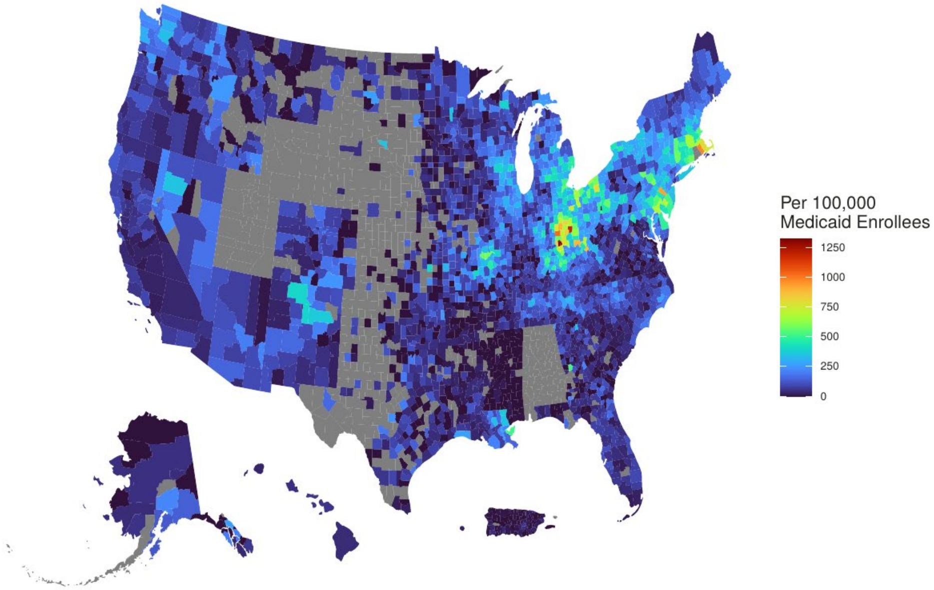

Source: Transformed Medicaid Statistical Information System (T-MSIS) Analytic Files (TAF), 2016-2018

© 2023 Lindner SR et al. *JAMA Health Forum.*

eTable 1. Overall and state-level prevelance levels of OUD diagnosis, OUD medication, and non-fatal overdoses

State	OUD	Any OUD medication	Non-fatal overdose
**Alaska**	3.9	51.3	3.9
**Arizona**	2.5	46.7	4.6
**Arkansas**	0.6	22.0	3.3
**California**	1.0	60.1	5.0
**Colorado**	2.4	40.7	5.4
**Connecticut**	6.3	77.2	6.3
**Delaware**	9.5	59.2	6.4
**District of Columbia**	2.5	57.6	2.9
**Florida**	1.2	39.4	5.6
**Georgia**	1.8	19.2	3.1
**Hawaii**	1.5	42.5	3.3
**Idaho**	2.8	28.7	5.2
**Illinois**	2.4	--	10.5
**Indiana**	3.0	63.6	6.9
**Iowa**	1.2	41.4	6.1
**Kansas**	1.6	17.7	4.0
**Kentucky**	6.8	53.1	4.8
**Louisiana**	2.2	37.4	6.4
**Maine**	8.0	82.8	2.1
**Maryland**	9.7	68	2.7
**Massachusetts**	6.3	78.6	9.4
**Michigan**	3.3	40.2	9.3
**Minnesota**	2.8	56.3	3.8
**Mississippi**	1.7	28.7	0.3
**Missouri**	3.0	45.1	7.1
**Montana**	3.0	60.7	2.6
**Nebraska**	0.9	31.0	5.6
**Nevada**	3.1	26.5	5.0

© 2023 Lindner SR et al. *JAMA Health Forum.*

**New Hampshire**	6.5	74.0	4.8
**New Jersey**	4.5	50.4	7.7
**New Mexico**	3.7	68.5	3.9
**New York**	3.2	36.6	5.4
**North Carolina**	3.2	44.4	3.2
**North Dakota**	1.4	26.8	4.9
**Ohio**	6.0	61.1	8.7
**Oklahoma**	2.9	19.0	2.3
**Oregon**	3.1	61.4	3.8
**Pennsylvania**	6.5	64.8	6.0
**Puerto Rico**	0.6	54.2	2.1
**South Carolina**	0.9	39.4	4.9
**South Dakota**	0.8	29.7	10
**Tennessee**	3.0	24.8	4.1
**Texas**	1.5	33.1	3.9
**Vermont**	4.8	82.0	2.3
**State**	**OUD**	**Any OUD medication**	**Non-fatal overdose**
**Virginia**	1.8	60.4	3.1
**Washington**	4.7	57.5	4.9
**West Virginia**	7.4	67.4	3.4
**Wisconsin**	4.0	68.7	5.6
Prevalence across all member-year observations	***3.0***	***55.2***	***5.8***

© 2023 Lindner SR et al. *JAMA Health Forum.*

eTable 2. Distribution of outcomes (restricted sample)

	OUD	Any OUD medication	Bupre-norphine	Meth-adone	Naltre-xone (oral)	Naltre-xone (extended release)	Overdoses (non-fatal)
**Mean**	2.73	59.99	31.45	27.02	3.38	3.61	6.03
**Minimum**	0.27	0.00	0.00	0.00	0.00	0.00	0.00
**1st quartile**	1.31	32.40	18.98	0.00	0.23	0.00	1.65
**Median**	2.24	47.06	30.30	5.12	2.27	0.19	3.60
**3rd quartile**	4.03	60.46	43.55	20.25	4.89	2.93	5.82
**Maximum**	21.74	87.50	87.50	82.27	26.32	29.42	25.00
**Extremal ratio**	80.90	---	---	---	---	---	---
**Interquartile range**	2.71	28.06	24.58	20.25	4.67	2.93	4.17
**Coefficient of variation**	87.38	25.13	50.98	70.10	79.85	112.19	49.41
**County median odds ratio**	1.68	1.66	1.74	2.96	1.55	1.87	1.50
**State median odds ratio**	1.78	1.97	1.74	18.90	2.37	4.79	1.79

© 2023 Lindner SR et al. *JAMA Health Forum.*

eReferences

1. Kaiser Family Foundation. Medicaid Behavioral Health Services: Methadone for Medication Assisted Treatment. Accessed: 04/05/2023. https://www.kff.org/other/state-indicator/medicaid-behavioral-health-services-methadone-for-medication-assisted-treatment-mat

2. Medicaid and CHIP Payment and Access Commission (MACPAC). Report to Congress on Medicaid and CHIP. Published 2018. Accessed: 04/05/2023. https://www.macpac.gov/wp-content/uploads/2018/06/June-2018-Report-to-Congress-on-Medicaid-and-CHIP.pdf

3. Miller N. Comprehensive Update on State Medicaid Coverage of Medication-Assisted Treatments and Substance Use Disorder Services. Published 2019. Accessed: 04/05/2023. https://www.rsat-tta.com/Files/MAT-Medicaid-Review_updated-12-2019

4. Substance Abuse and Mental Health Services Administration (SAMHSA). Medicaid Coverage of Medication-Assisted Treatment for Alcohol and Opioid Use Disorders and of Medication for the Reversal of Opioid Overdose. Published 2018. Accessed: 04/05/2023. https://store.samhsa.gov/sites/default/files/d7/priv/medicaidfinancingmatreport_0.pdf

5. Medicaid.gov. DQ Atlas. Accessed: 04/05/2023. https://www.medicaid.gov/dq-atlas/

© 2023 Lindner SR et al. *JAMA Health Forum.*
