# Supplementary material for: State- and County-Level Geographic Variation in Opioid Use Disorder, Medication Treatment, and Opioid-Related Overdose Among Medicaid Enrollees
Source: JAMA Health Forum. 2023 Jun 23;4(6):e231574. doi: 10.1001/jamahealthforum.2023.1574 (PMC10290243; doi:10.1001/jamahealthforum.2023.1574)
Supplement: Supplement 2. — Data Sharing Statement [file jamahealthforum-e231574-s002.pdf]

Data Sharing Statement

Lindner. State- and County-Level Geographic Variation in Opioid Use Disorder, Medication Treatment, and Opioid-Related Overdose Among Medicaid Enrollees. *JAMA Health Forum*. Published June 23, 2023. doi:10.1001/jamahealthforum.2023.1574

Data
Data available: No

Additional Information
Explanation for why data not available: The data for the analysis is not publicly available.
